# Evidence for Early European Neolithic Dog Dispersal: New Data on Southeastern European Subfossil Dogs from the Prehistoric and Antiquity Ages

**DOI:** 10.3390/genes10100757

**Published:** 2019-09-26

**Authors:** Iskra Yankova, Miroslav Marinov, Boyko Neov, Maria Petrova, Nikolai Spassov, Peter Hristov, Georgi Radoslavov

**Affiliations:** 1 Department of Animal Diversity and Resources, Institute of Biodiversity and Ecosystem Research, Bulgarian Academy of Sciences, 1040 Sofia, Bulgaria; kikoicko18@abv.bg (I.Y.); maff97@abv.bg (M.M.); boikoneov@gmail.com (B.N.); peter_hristoff@abv.bg (P.H.); 2 Department of Structure and Function of Chromatin, Institute of Molecular Biology, Bulgarian Academy of Sciences, 1040 Sofia, Bulgaria; mhristova84@abv.bg; 3 Palaeontology and Mineralogy Department, National Museum of Natural History, Bulgarian Academy of Sciences, 1040 Sofia, Bulgaria; nspassov@nmnhs.com

**Keywords:** ancient DNA, population structure, dog, the Balkans

## Abstract

The history of dog domestication is still under debate, but it is doubtless the process of an ancient partnership between dogs (*Canis familiaris*) and humans. Although data on ancient DNA for dog diversity are still incomplete, it is clear that several regional dog populations had formed in Eurasia up to the Holocene. During the Neolithic Revolution and the transition from hunter-gatherer to farmer societies, followed by civilization changes in the Antiquity period, the dog population structure also changed. This process was due to replacement with newly formed dog populations. In this study, we present for the first time mitochondrial data of ancient dog remains from the Early Neolithic (8000 years before present (BP)) to Late Antiquity (up to 3th century AD) from southeastern Europe (the Balkans). A total of 16 samples were analyzed, using the mitochondrial D-loop region (HVR1). The results show the presence of A (70%) and B (25%) clades throughout the Early and Late Neolithic Period. In order to clarify the position of our results within the ancient dog population in Eneolithic Eurasia, we performed phylogenetic analysis with the available genetic data sets. This data showed a similarity of the ancient Bulgarian dogs to Italian (A, B, and C clades) and Iberian (clades A and C) dogs’ populations. A clear border can be seen between southern European genetic dog structure, on the one hand, and on the other hand, central-western (clade C), eastern (clade D) and northern Europe (clades A and C). This corresponds to genetic data for European humans during the same period, without admixture between dog populations. Also, our data have shown the presence of clade B in ancient Eurasia. This is not unexpected, as the B haplogroup is widely distributed in extant Balkan dogs and wolves. The presence of this clade both in dogs and in wolves on the Balkans may be explained with hybridization events before the Neolithic period. The spreading of this clade across Europe, together with the A clade, is related to the possible dissemination of newly formed dog breeds from Ancient Greece, Thrace, and the Roman Empire.

## 1. Introduction

In recent years, it has been increasingly assumed that dog domestication was a very early process that began at the end of the Pleistocene. Dog domestication is apparently linked to the gradual synanthropy of wolf populations, as a result of commensal relations between man and wolf at the end of the late Pleistocene [1,2]. The first data from dog-like remains came from the Razboinichya Cave in the Altai Mountains of Siberia (33,000 years before present (BP)) and the Goyet cave in Belgium (c. 31,700 BP) [3,4]. The first remains that can be confidently assigned to dogs date from 15,000 years ago in Europe and 12,500 years ago in East Asia [5,6]. Genetic studies of these remains have not shown any similarity to recent wolves or dogs [3,7]. The Pleistocene domestication cannot be accepted as absolutely certain, given the large morphological variability of Pleistocene wolves [7]. Since then, the main questions have been related to place and time of origin, domestication, and the influence of hybridization events between domesticated dog and local wolf populations.

According to mitochondrial DNA (mtDNA) studies, there are six main clades, assigned A, B, C, D, E, and F, as well as many sub-clades, characterizing dog populations in the world [8,9,10]. Clades A, B, and C are the most widely distributed (95.9%) among recent dog populations, while D, E, and F have regional geographic distribution. For example, in West Eurasia, the sub-haplogroup A1 displays a frequency of about 70%, while clades B and C are present with frequencies of 20% and 10%, respectively [8,10]. In the Middle East, this proportion is almost the same, but with more presented sub-haplogroups [11].

There is no data concerning the analysis of ancient dog DNA in some geographical regions. Despite this, available data reveal quite different population structures compared to present day dogs. These alterations are due to the emergence of the first civilizations, as well as dogs’ population admixture, population replacement, and population movement. For example, pre-Columbian American dogs were identical to East-Eurasian (Siberian) dogs, which are nowadays mixed with West-Eurasian dogs, after the Age of Discovery (15th century) [5,6,12]. Similarly, in the Pacific, including the islands of Polynesia, a post-Lapita dog introduction from southern Island Southeast Asia has been suggested [12,13].

### 1.1. Ancient Dog Populations from Europe

**Figure 1:**
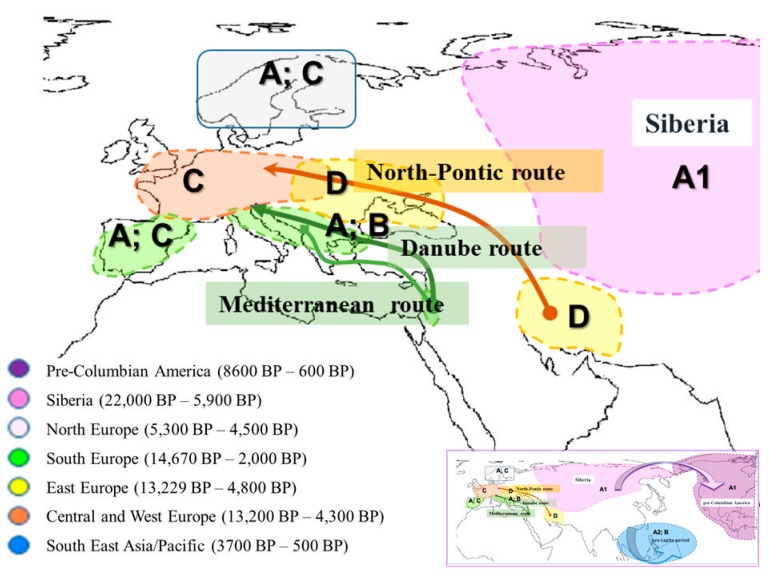
Population structure of ancient dogs worldwide, based on mitochondrial DNA (mtDNA) data. A world map showing the distribution of main haplogroups in ancient dogs. The main migration routes are also shown.

One of the most investigated regions concerning ancient dog mtDNA is Europe. Up to now, most studies have shown that old Europe (from the Pleistocene to the Holocene) may be divided into at least three or four regions of dog populations, based on mtDNA analysis. One of them is Central and West Europe, including France, Germany, Switzerland, and Hungary. Typical for these parts of Europe is the prevalence of clade C (over 80%) mixed with clade D [5,6,14,15,16,17,18,19] (Figure 1). The second region includes Eastern Europe (Romania, Moldova, Ukraine), up to Iran and the Middle East. The basic clade in these regions is D (over 90%), and there are traces of A and C [5,6,15,16]. In Northern Europe (Scandinavia and Estonia) there are clades A and C [5,16,17]. Furthermore, clade D, the most common clade currently found in Scandinavia, is missing [8,20,21]. There have been only two studies from southern Europe concerning ancient dog populations [22,23]. The first includes only five samples (three wolves and two dogs) from the Apennines. The obtained results show the presence of clades A, B, and C [5,16,22]. The study of the Mesolithic Iberian dog found a high frequency of clade A (83%) and clade C (17%). This is the first report of the prevalence of dog haplogroup A in pre-Neolithic Europe. Most of the authors have suggested that clade A is associated with Neolithic farmer migration in ancient Europe from the Middle East, while clade B probably originated from southeastern Europe (the Balkans and the Apennines), because of the high frequency of extant Balkan wolves with similar haplotypes [5,22,24,25,26].

All these data from pre-historic Europe have shown a dramatic difference between ancient and present-day dog populations. This change may be explained by the replacement of local dogs with more improved breeds from the southern part of Europe, the Mediterranean region, and the Near East during Late Antiquity and the Medieval age [22].

### 1.2. Balkan Neolithic and Chalcolithic Periods: Specifics of Human Societies and Understanding of Early European Farmer Migration Routes from the Near East

Southeastern Europe includes the Balkan Peninsula and the Apennines. These geographical regions are important points concerning human history and migration processes, as well as the accompanying domesticated animals and the crops from the Near East. It is well known that during the Last Glacial Maximum (22,000–14,000 BP), the sea level was about 125 m lower than nowadays [27]. Therefore, large territories of land were connected. For example, the Balkan Peninsula was connected terrestrially to Anatolia via the Sea of Marmara in the east, and also to a large part of the Apennines via the Adriatic Sea in the west [22]. This enormous contact zone was important for the free movement of human societies, but also the movement of wild animal populations during the Mesolithic and the Early Neolithic periods.

Therefore, Early Neolithic cultures from the Fertile Crescent and Anatolia were directly connected to southeastern Europe (especially during the Late Neolithic period, 8000 BP). During this period, three migration routes for the dissemination of Neolithic farmers into Europe existed: the Mediterranean, the Balkans (the Danube River), and the North Pontic steppes (Figure 1).

The most recent study on human population from the Mesolithic to the Iron Age has revealed that human populations in the Balkans (southeastern Europe) and in Anatolia shared a similar genetic profile in contrast with North Pontic Steppe Neolithic humans, as well as those in central and western Europe [28]. Despite the similarity of the Eastern and the Southeastern Neolithic and Chalcolithic cultures, these genetic data suggest that three migration routes (the Mediterranean, the Danubean, and the North Pontic) existed for the dispersal of Neolithic farmers from the Fertile Crescent. The data of this research clearly shows human migrations from the north to east direction in the Balkans during the Chalcolithic and the Bronze Ages, but also the dissemination of southeastern human farmers into the Central Europe region (LBK culture: Austria, 5100–5000 BC). This evidence also suggests different migration routes for livestock, such as cattle, goats, and sheep [29].

In the Neolithic to the Chalcolithic Age, livestock farming on the Balkans was related to intensive cattle, goat, and sheep breeding [30,31,32], with a prevalence of cattle (over 30%) in the North Balkans and the domination of sheep breeding in the south. This animal husbandry was accompanied everywhere by the presence in the studied settlements of ancient races of dogs.

The processes of domestication and dog breed creation continued later, up to the Early and the Late Antique periods. At this time, the first data on dog breeds on the Balkans were described by Aristoteles [33], and later, during the Roman empire, by Xenophon (*Cynegeneticus*, 2000 years ago). The authors commented on dozens of different dog breeds, including guard, hunting, and companion dogs.

Our study tries to enrich data for ancient southeastern Europe—in particular, the Balkan Peninsula (Bulgarian) dogs, based on mtDNA (partial D-loop region, HVR1) analysis. We have performed phylogenetic analysis to compare our results with other ancient as well as recent European dog and wolf populations.

## 2. Materials and Methods

### 2.1. Archeological Sample Collection

Twenty-five samples (bones and dental material) from ancient dogs from the collection of NMNH-BAS were studied from several archaeological sites: Early Neolithic (8500–7500 BP; Gradeshnitsa/Malo Pole, Ohoden–Valoga Slatina, Sofia district); Late Neolithic (7500–7000 BP; Topolnica, Promachon, and Budzhaka-Sozopol); Early Chalcolithic (6950–6500 BP; Okol-Glava, Gniljane, Sultan (Nevski) Popovo, and settlement mound Burgas); Late Chalcolithic (6500–6000 BP; Dolnoslav, Varna); Bronze (6000–5000 BP; Urdoviza–Kiten, Baley); Late Antiquity (Kapitan Andreevo, Dyadovo village–Nova Zagora, Charda village, Yambol district, Trakia motorway, Yambol district, and Academic, Plovdiv) (Table S1 and Figure S1).

The *Canis familiaris* remains were determined and assigned to two primitive prehistoric morphotypes (and probable crossbreeds between them), representing the two main Mesolithic/Neolithic–Chalcolithic canine races of the Balkans, which can be called conditionally *Canis familiaris “intermedius*” and *C. f. “palustris*”. The mesolithic form *C. f. vlasac*, described from Serbia [34,35], is not different from *C. f. intermedius*, and represents a slightly younger form of the latter, with skull/tooth dimensions that are in general within the lower values of the individual variations of *C. f. vlasac*. *C. f.” palustris*,” however, is the more domesticated small form [32].

### 2.2. Ancient DNA Isolation

Due to the special features of working with ancient DNA (aDNA) in the laboratories of the Institute of Biodiversity and Ecosystem Research (IBER-BAS) and the Institute of Molecular Biology (IMB-BAS), certain conditions were created to prevent contamination with exogenous DNA and to ensure the authenticity of the results. All experiments were performed according to standard precautions in specialized and geographically distinct laboratories for aDNA work: bone material processing, DNA isolation, and PCR amplification [36,37]. Briefly, this included establishing independent laboratories (premises and buildings) for working with ancient DNA, treating surfaces and solutions with UV radiation (45 W, 72 h), heat treatment (over 180 °C, 12 h), acid treatment (2.5 M HCl, 48 h), and sodium hypochlorite (40%, 48 h), as well as washing with ultrapure water and air filtration in the premises. Consumables and reagents were licensed to work with human DNA.

The genetic material was isolated according to the protocol of Yang et al., with minor modifications [38,39]. To prevent contamination of the bone surface by foreign DNA, the samples were treated sequentially with sodium hypochlorite (40%) and 2% hydrochloric acid, then were washed with ultrapure H_2_O several times. After drying for 24–48 h in UV-irradiation medium and constant air filtration, the surface layer was removed, and then bone powder was obtained by Dremel (Grinding A 11 basic analytical mill, Germany), which was further homogenized in metal mortars. Treatment of bone material for isolation of the aDNA was performed from 400–500 mg of bone powder dissolved in 5 mL of lysis buffer (0.5 M EDTA, 2% Sodium dodecyl sulfate, 0.1 M Tris pH8, 10 μL/mL Mercaptoethanol, 20 μL/mL Proteinase K). The samples were incubated in a hybridizer (Hybridiser HB-2D, Techne, Cambridge, United Kingdom) at constant rotation at 56 °C for 36–48 h. The samples were centrifuged at 5000 rpm for one hour, after which the supernatant was filtered through 0.45 μm filters and transferred to 15 ml tubes. DNA isolation was performed by silicone membrane technology, including the use of DNA isolation columns (GeneMatrix, E3520, EURx, Gdańsk, Poland), 5 M GuSCN (Sigma-Aldrich, Taufkirchen, Germany) binding reagent (*v*/*v*), and 20% EtOH. The aDNA bound to the silica columns was purified twice with a 70% ethanol wash solution and dissolved in ultrapure water. The isolated aDNA was stored at −20 °C.

### 2.3. PCR Amplification and Sequencing

We used a nested PCR reaction to amplify three overlapping fragments of the HVRI region. The PCR products of the second nested PCR primer sets were 15,527–15,703 bp (177 bp) and 15,433–15,703 bp (270 bp), 15,680–15,804 bp (125 bp), and 15,781–16,090 bp (310 bp). The position of the primers is based on the dog reference sequence NC_002008 [40]. The primers for amplification are listed in Table S2. For the negative control, we used primer sets from the first nested PCR reaction, with and without template DNA.

All PCR reactions were performed with 10 ng/μL DNA in a final volume of 50 μL, by using NZYTaq Colourless Master Mix (Cat No–MB040, NZYTech, Lisbon, Portugal).

PCR reactions were performed under the following conditions: initial denaturation at 94 °C for 5 min; 40 cycles of denaturation at 94 °C for 30 s, primer hybridization at −50 °C for 30 s, elongation at 72 °C for 1 min, and final elongation at 72 °C for 10 min. The amplified fragments were separated and visualized on 2% agarose gel electrophoresis.

The successfully amplified products were purified by a PCR purification kit (Gene Matrix, PCR clean-up kit, EURx, Poland) and sequenced in both directions by a PlateSeq kit (Eurofins Genomics, Ebersberg, Germany).

### 2.4. Phylogenetic Reconstruction

The obtained sequences were manually edited and aligned by MEGA software version 7.0 [41], using the dog mtDNA sequence NC_002008 [40] and EU789787 [9] as references. Sequences were analyzed by polymorphic SNPs, and haplogroups were determined according to [10,42], as well as the MitoToolPy program [43] (http://www.mitotool.org/mp.html) with reference sequence EU789787 [9]. The phylogenetic analysis was based on the archaeological dog samples used in this study, as well as on all available ancient DNA dog sequences in GenBank and the Dome tree [5,6,12,13,15,16,17,22,31,32,33,44] (Table S3). Ancient and recent wolves [6,22,24,25,26,45], as well as the recent Bulgarian native dog [46], (Table S3) were characterized using network analysis NETWORK 4.5.1.6 (Fluxus Technology Ltd.; available at http://fluxusengineering.com).

In order to graphically display (and summarize) the mitochondrial relationships among the analyzed ancient dog populations and all ancient samples available in GenBank, we performed a principal component analysis (PCA)—a method that considers each haplogroup as a discrete variable and allows a summary of the initial dataset into principal components (PCs). Principal component analysis (PCA) was performed using Excel software implemented by XLSTAT, as described elsewhere [47]. Two PCAs were carried out, one by considering only our sample, and the other by including the available ancient dog mtDNA records obtained from GenBank and the Dome tree (Table S3). PCA is a widely used dimension reduction method that seeks to explain the variance of multivariate data by a smaller number of variables (the principal components, or PCs), which are linear functions of the original variables—in this case, the haplogroup frequencies. The haplogroup frequencies used as source data for the PCA were calculated by considering only different haplogroups (Table S4). The rarest haplogroups were phylogenetically grouped and among the large plethora of available data; only those represented by different haplogroups were included in the analysis, in order to increase the statistical significance.

The obtained sequences included in this study were deposited in the National Center for Biotechnology Information (NCBI) GenBank database, under accession numbers NCBI: MH937186–MH937206.

## 3. Results

### 3.1. Phylogenetic Analysis and Haplogroup Classification

Ancient DNA was successfully amplified in 21 out of 25 samples tested. Out of the 21 sequences, 16 were used for phylogenetic analysis, as they covered informative sites for haplogroups assignment. Eleven sequences were assigned to clade A (68.7%), four to clade B (25.0%), and one to clade D (6.2%). The distribution of clade A and B frequencies were, respectively, 83.3%/16.7% (Neolithic), 60.0%/40.0% (Chalcolithic), 66.7%/33.3% (Bronze), and 50.0%/33.3% (Antiquity). In contrast, clade D, highly dispersed in ancient East Europe, was found only in one sample from the Late Antiquity period (16.7%; Table S1). These sequences have informative sites from position 15595 bp to 15784 bp (189 bp), and include the first region of *HVR1*, according to reference sequence EU789787 [9]. We analyzed only this region, because most of the ancient dog sequences available in GenBank also used this region as the most informative one for haplogroup assignment. Four of our unassigned sequences have only the third amplified fragment of *HVR1* (Tables S1 and S3).

### 3.2. Comparative Analyses of Ancient Dog Haplotypes and Bulgarian Samples

**Figure 2:**
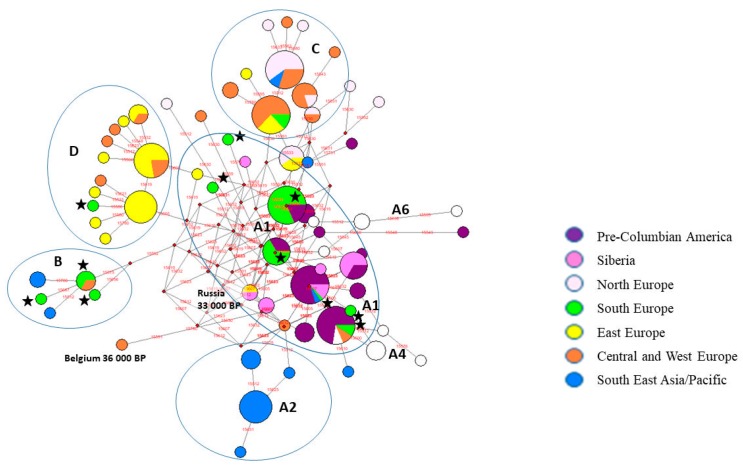
The reduced median network of the main mtDNA haplotypes from ancient dogs of different regions of the world. Bulgarian ancient dog haplotypes are shown with asterisks.

**Figure 3:**
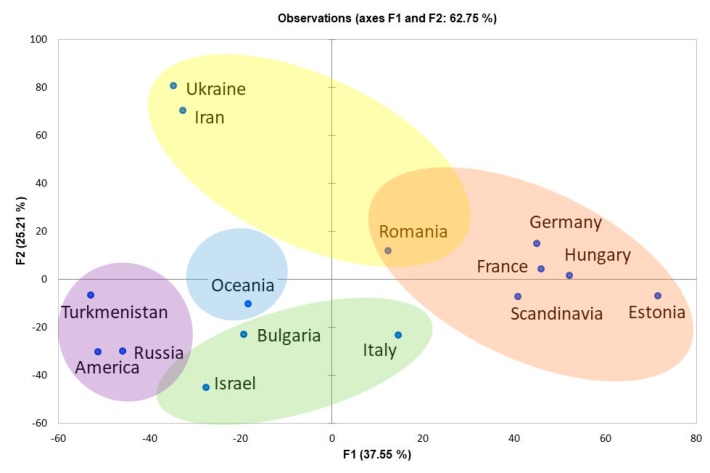
Principal component analysis of the main mtDNA haplotypes from ancient dogs of different regions of the world.

About 230 ancient dog sequences available in GenBank and the Dryad Digital Repository package [16] were explored by reduced-median-network analysis. In this analysis, the ancient dog samples were from ancient Eurasia, pre-Columbian America, and the Pacific pre-Lapita period (Figure 2, Table S3). All haplotypes were separated into four main clades: A, B, C, and D. There is a clear geographical differentiation between the distribution of C and D clades, which are specific for central, western, and eastern Europe. Clade A is split into many sub-clades—i.e., A2 for Southeast Asia and the Pacific; A1 for Siberia, Central Asia, and pre-Columbian America; and A4 and A6 for northern Europe (Scandinavia). Clades A and B are also characteristic for the Mediterranean region (Italy, Bulgaria, and Israel). Similarly, PCA analysis grouped all the ancient samples into five distinct groups (Figure 3, Table S4).

### 3.3. Comparative Analysis Among Ancient and Recent Balkan Dogs as well as Recent Gray Wolf Haplotype Distribution

**Figure 4:**
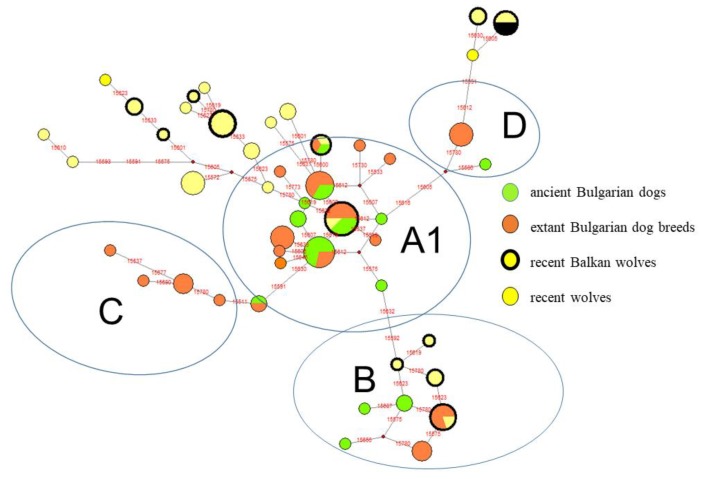
The reduced median network of the main mtDNA haplotypes from ancient and recent Balkan dogs and wolves.

About 120 sequences, including recent Balkan dogs and wolf populations, as well as ancient Bulgarian and Italian samples, were analyzed by a median-joining network (Figure 4, Table S3). All samples (dogs and wolves) were explored as described previously, from position 15,595–15,784 bp (189 bp) of the first amplified region of *HVR1*, according to reference sequence EU789787 [9]; haplotypes were determined according to the classifications of [10] and [42], as well as the MitoToolPy program [43]. The results show that most of the ancient and recent dog haplotypes from clades A and B are similar to recent Balkan wolf haplotypes. The Balkan wolf haplotypes are not grouped into C and D clades. Moreover, there are wolf haplotypes that do not include present-day dog haplotypes.

## 4. Discussion

### 4.1. Clade A: Specific for Neolithic Southern Europe (the Mediterranean Region)

Prehistoric Neolithic Balkan dogs were primitive in terms of stage of domestication, as their morphological features were relatively close to ancestral wolf morphology, such as that of *C.f. vlasac* [32,33,34]. Our data have shown a prevalence of A and B dog clades from the Neolithic to the Antique period. During this time, the proportion of these clades was preserved, with minor changes. Despite the small size of the investigated samples, these results are in direct contrast with the border regions in the northern direction, where the Danube River serves as a border region. The investigated ancient dog samples from Romania, Moldova, and Ukraine (9000–5000 BP) showed a prevalence of C and D clades [16] (Table S3).

Otherwise, our data correlate with ancient samples from Italy and Israel, where, despite the small size of samples, a prevalence of clades A and C for Italy has been revealed [6,15,16,22]. These data, even insufficient, have suggested that A clade dog populations replaced old Central European ones. It is possible that this genetic profile is associated with the Fertile Crescent and Anatolian ancient dogs. Adding to this hypothesis is an investigation of the Iberian pre-Neolithic dogs, where clades A (with high frequency 83%) and C are also reported [23]. This and another study describe haplotypes A, B, and C in Mesolithic wolves that are shared with dogs [22,23]. The authors cannot exclude local/independent processes for the domestication of dogs carrying haplogroups A and C, because Mesolithic wolves of the same region and time period also shared identical haplotypes (Figure 1).

### 4.2. Clade B: The Possibility of a Southeastern European (Balkans) Origin of Dogs from Local Wolf Predecessors

In modern dogs, clade B is widely distributed, with a frequency of over 20% [8,10]. This clade consists of two sub-clades: B1 and B2. The B1 sub-clade is disseminated worldwide, with a frequency of about 21%, while the B2 sub-clade has a regional distribution, mainly in Eastern Asia, with a frequency of about 10% [10,48]. Although there is a high frequency of clade B in modern dogs, in ancient samples until now these sub-haplogroups have been observed very rarely, with a frequency of about 1%. There is one sample in France and Turkmenistan, and there are a few samples from Southeastern Asia and Oceania [13,15,17]. In our sample sets, the B1 sub-clade has a high frequency (over 20%). This haplogroup is common among the Neolithic, the Chalcolithic, and the Bronze Age samples, but is also present in Antiquity with almost equal frequency.

These data are not very surprising, because clade B is widely and unusually distributed in recent Balkan wolf populations, identified in several studies [6,24,25,26,46]. These studies have proposed that clade B possibly originated from the Balkans, although there are some cases of B-type wolves worldwide. In a previous study of Bulgarian native dogs, clade B was observed with about 20% frequency [47].

The only investigation about ancient dogs and wolves concerning southern Europe was carried out by Verginelli et al. [22]. In this study, the authors investigated three wolves or large canids from 14,000–10,000 years ago, as well as two medium-sized dogs from 4000 years ago. One of the wolves’ samples was assigned as clade B (PIC-2), while others belonged to A (PIC-3) and C (PIC-1) clades (Figure 4). The authors hypothesized about the possible origin of B clades from the Balkans, due to the high frequency of B-type Balkan wolves. In addition, the authors determined that in southeastern Europe (the Balkans and the Apennines), there is a high frequency of clade B in the gray wolf population. The discovery of clade B in the primitive dog race (*intermedius*–*vlasac* morpho-group) from the Early Neolithic of Slatina (Sofia) presents new arguments in favor of the morphologically [34,35] and genetically [22] argued hypothesis that some prehistoric to recent dog races (races which have the B-clade) originated on the Balkans.

### 4.3. Population Structure of Ancient Dogs from the Neolithic Period

The question of dog origin is geographically, genetically, and archaeologically complex. Ancient DNA analysis from relevant areas of the world should allow a better understanding of the evolution of dogs from their predecessor: the gray wolf. In this respect, there are a few hypotheses about dog domestication and spread. Based on recent mtDNA haplotype distributions, the common opinion is that in the center of domestication, the genetic diversity is the highest; thus, there must be a high level of various haplotypes from different clades. Due to this reason, the East Asian and the Near Eastern (Anatolian and Fertile Crescent) centers were proposed for dog origin centers [9,11]. The migration routes from the centers of domestication are characterized by a bottleneck type of dissemination, which is unique for bordered regions worldwide, such as Europe, North Africa, the Pacific Islands. etc. For example, the genetic profile of recent European dogs is characterized by the prevalence with various A1 and B1 sub-clades [10,18,48], while dogs in Southwest Asia (Anatolia and Fertile Crescent) have a mixed profile with other A and B sub-clades [11]. Recent European C1, D1, and D2 sub-clades are considered to be regionally specific sub-clades, and as a “relic” from ancient times of dog–wolf hybridization—i.e., modern populations are very rarely representative of ancient populations [8,9].

In contrast to molecular data about recent dogs, evidence for ancient dogs, though still incomplete, shows a homogeneous and simple genetic structure of prehistory dog populations (Figure 1). In western, central, and northern Europe, there is a prevalence of the C clade; in eastern Europe and the Middle East (Iran), of clade D; in Southeastern Asia, Australia, and the Pacific Islands, there is a prevalence of the specific sub-clade A2; and in Siberia, Central Asia (Turkmenistan), and pre-Columbian America, a prevalence of specific A1 sub-clades [5,6,12,13,14,15,49,50] (Figure 2). Key geographic regions for understanding dog history, such as southern Europe and the Near East, North Africa, and ancient central and southern Asia (China and India), are still uninvestigated.

## 5. Conclusions

In conclusion, our data have enriched the information about ancient dogs’ structure, especially in southeastern Europe. The results from the Neolithic, Chalcolithic, and Antiquity periods in Bulgaria, have demonstrated the dominance of A (70%) and B (25%) dog clades, as well as homogenic structures of these haplogroups throughout the whole investigated period; however, they have also demonstrated an absence of clade C and only one case of D clade (late Antiquity period). These data have revealed the similarity of the Bulgarian dogs’ structure to that of ancient Italian dogs (A, B, and C clades). Our data have shown for the first time the presence of clade B in ancient Eurasia. This is not unexpected, because of the fact that the B haplogroup was widely distributed in extant Balkan wolves and dogs. The presence of this clade in both wolves and dogs on the Balkans may be explained with hybridization events before the Neolithic period. The spreading of this clade across Europe, together with the A clade, is related to the possible dispersal dissemination of newly formed dog breeds.

## Supplementary Materials

The following are available online at https://www.mdpi.com/2073-4425/10/10/757/s1, Figure S1: Map showing sample locations. Table S1: Archaeological samples. Table S2: Primers used for amplification. Table S3: Ancient dog samples used in phylogenetic analysis. Table S4: Source data for the PCA analysis of ancient dog mtDNA haplogroups.

## References

[1] Sullivan J.O., Hall R.L., Sharp H.S. (1978). Variability in the wolf, a group hunter. Wolf and Man.

[2] Morey D.F., Jeger R. (2017). From wolf to dog: Late Pleistocene ecological dynamics, altered trophic strategies, and shifting human perceptions. Hist. Biol..

[3] Ovodov N.D., Crockford S.J., Kuzmin Y.V., Higham T.F., Hodgins G.W., van der Plicht J. (2011). A 33,000-year-old incipient dog from the Altai Mountains of Siberia: Evidence of the earliest domestication disrupted by the Last Glacial Maximum. PLoS ONE.

[4] Germonpré M., Sablin M.V., Stevens R.E., Hedges R.E., Hofreiter M., Després V.R. (2009). Fossil dogs and wolves from Palaeolithic sites in Belgium, the Ukraine and Russia: Osteometry, ancient DNA and stable isotopes. J. Archaeol. Sci..

[5] Frantz L.A., Mullin V.E., Pionnier-Capitan M., Lebrasseur O., Ollivier M., Perri A., Tresset A. (2016). Genomic and archaeological evidence suggest a dual origin of domestic dogs. Science.

[6] Thalmann O., Shapiro B., Cui P., Schuenemann V.J., Sawyer S.K., Greenfield D.L., Napierala H. (2013). Complete mitochondrial genomes of ancient canids suggest a European origin of domestic dogs. Science.

[7] Boudadi-Maligne M., Escarguel G. (2014). A biometric re-evaluation of recent claims for Early Upper Palaeolithic wolf domestication in Eurasia. J. Archaeol. Sci..

[8] Savolainen P., Zhang Y.P., Luo J., Lundeberg J., Leitner T. (2002). Genetic evidence for an East Asian origin of domestic dogs. Science.

[9] Pang J.F., Kluetsch C., Zou X.J., Zhang A.B., Luo L.Y., Angleby H., Ardalan A., Ekström C., Sköllermo A., Lundeberg J. (2009). mtDNA data indicate a single origin for dogs south of Yangtze River, less than 16,300 years ago, from numerous wolves. Mol. Biol. Evol..

[10] Duleba A., Skonieczna K., Bogdanowicz W., Malyarchuk B., Grzybowski T. (2015). Complete mitochondrial genome database and standardized classification system for *Canis lupus familiaris*. Forensic Sci. Int. Genet..

[11] Ardalan A., Kluetsch C.F., Zhang A.B., Erdogan M., Uhlén M., Savolainen P. (2011). Comprehensive study of mtDNA among Southwest Asian dogs contradicts independent domestication of wolf, but implies dog–wolf hybridization. Ecol. Evol..

[12] Witt K.E., Judd K., Kitchen A., Grier C., Kohler T.A., Ortman S.G., Malhi R.S. (2015). DNA analysis of ancient dogs of the Americas: Identifying possible founding haplotypes and reconstructing population histories. J. Hum. Evol..

[13] Greig K., Gosling A., Collins C.J., Boocock J., McDonald K., Addison D.J., Liu F. (2018). Complex history of dog (*Canis familiaris*) origins and translocations in the Pacific revealed by ancient mitogenomes. Sci. Rep..

[14] Deguilloux M.F., Moquel J., Pemonge M.H., Colombeau G. (2009). Ancient DNA supports lineage replacement in European dog gene pool: Insight into Neolithic southeast France. J. Archaeol. Sci..

[15] Pionnier-Capitan M. (2010). La Domestication du Chien en Eurasie: Étude de la Diversitémpassée, Approches Ostéoarchéologiques, Morphométriques et Paléogénétiques. Ph.D. Thesis.

[16] Ollivier M., Tresset A., Frantz L.A., Bréhard S., Bălăşescu A., Mashkour M., Bartosiewicz L. (2018). Dogs accompanied humans during the Neolithic expansion into Europe. Biol. Lett..

[17] Malmström H., Svensson E.M., Gilbert M.T., Willerslev E., Götherström A., Holmlund G. (2007). More on contamination: The use of asymmetric molecular behavior to identify authentic ancient human DNA. Mol. Biol. Evol..

[18] Botigué L.R., Song S., Scheu A., Gopalan S., Pendleton A.L., Oetjens M., Bobo D. (2017). Ancient European dog genomes reveal continuity since the Early Neolithic. Nat. Commun..

[19] Skoglund P., Ersmark E., Palkopoulou E., Dalén L. (2015). Ancient wolf genome reveals an early divergence of domestic dog ancestors and admixture into high-latitude breeds. Curr. Biol..

[20] Klütsch C.F., Seppälä E.H., Fall T., Uhlén M., Hedhammar Å., Lohi H., Savolainen P. (2011). Regional occurrence, high frequency but low diversity of mitochondrial DNA haplogroup d1 suggests a recent dog-wolf hybridization in Scandinavia. Anim. Genet..

[21] Adeola A.C., Ommeh S.C., Song J.J., Olaogun S.C., Sanke O.J., Yin T.T., Agwanda B.R. (2017). A cryptic mitochondrial DNA link between North European and West African dogs. J. Genet. Genom..

[22] Verginelli F., Capelli C., Coia V., Musiani M., Falchetti M., Ottini L., Mariani-Costantini R. (2005). Mitochondrial DNA from prehistoric canids highlights relationships between dogs and South-East European wolves. Mol. Biol. Evol..

[23] Pires A.E., Detry C., Chikhi L., Rasteiro R., Amorim I.R., Simões F., Cardoso J.L. (2019). The curious case of the Mesolithic Iberian dogs: An archaeogenetic study. J. Archaeol. Sci..

[24] Vilà C., Savolainen P., Maldonado J.E., Amorim I.R., Rice J.E., Honeycutt R.L., Wayne R.K. (1997). Multiple and ancient origins of the domestic dog. Science.

[25] Randi E., Lucchini V., Christensen M.F., Mucci N., Funk S.M., Dolf G., Loeschcke V. (2000). Mitochondrial DNA variability in Italian and East European wolves: Detecting the consequences of small population size and hybridization. Conserv. Biol..

[26] Koblmüller S., Vilà C., Lorente-Galdos B., Dabad M., Ramirez O., Marques-Bonet T., Leonard J.A. (2016). Whole mitochondrial genomes illuminate ancient intercontinental dispersals of grey wolves (*Canis lupus*). J. Biogeogr..

[27] Clark P.U., Dyke A.S., Shakun J.D., Carlson A.E., Clark J., Wohlfarth B., McCabe A.M. (2009). The Last Glacial Maximum. Science.

[28] Mathieson I., Alpaslan-Roodenberg S., Posth C., Szécsényi-Nagy A., Rohland N., Mallick S., Fernandes D. (2018). The genomic history of southeastern Europe. Nature.

[29] Lenstra J., Ajmone-Marsan P., Beja-Pereira A., Bollongino R., Bradley D., Colli L., Ginja C. (2014). Meta-analysis of mitochondrial DNA reveals several population bottlenecks during worldwide migrations of cattle. Diversity.

[30] Spassov N., Iliev N., Boev Z. (2001). Animal remains from the Eneolithic site near the village of Dolnoslav, Plovdiv District, South Bulgaria. Hist. Nat. Bulg..

[31] Spassov N., Iliev N. (2002). The animal bones from the prehistoric necropolis near Durankulak and the latest record of *Equus hydruntinus* Regalia (NE Bulgaria). Durankulak.

[32] Spassov N., Iliev N., Roodenberg J., Leshtakov K., Petrova V. (2014). CHAPTER XII. 1. Bone remains from domestic and wild animals. Yabalkovo.

[33] Kitchell K.F. (2014). Animals in the Ancient World from A to Z.

[34] Bökönyi S., Clason A. (1975). *Vlasac*: An early site of dog domestication. Archaeozoological Studies: Papers of the Archaeozoological Conference 1974.

[35] Dimitrijević VVuković S. (2015). Was the dog locally domesticated in the Danube Gorges? Morphometric study of dog cranial remains from four Mesolithic–Early Neolithic archaeological sites by comparison with contemporary wolves. Int. J. Osteoarchaeol..

[36] Pääbo S., Poinar H., Serre D., Jaenicke-Despres V., Hebler J., Rohland N., Hofreiter M. (2004). Genetic analyses from ancient DNA. Annu. Rev. Genet..

[37] Willerslev E., Cooper A. (2005). Ancient DNA. Proc. Biol. Sci..

[38] Yang D.Y., Eng B., Waye J.S., Dudar J.C., Saunders S.R. (1998). Technical note: Improved DNA extraction from ancient bones using silica-based spin columns. Am. J. Phys. Anthropol..

[39] Hristov P., Spassov N., Iliev N., Radoslavov G. (2017). An independent event of Neolithic cattle domestication on the South-eastern Balkans: Evidence from prehistoric aurochs and cattle populations. Mitochondrial DNA.

[40] Kumar S., Stecher G., Tamura K. (2016). MEGA7: Molecular evolutionary genetics analysis version 7.0 for bigger datasets. Mol. Biol. Evol..

[41] Kim K.S., Lee S.E., Jeong H.W., Ha J.H. (1998). The complete nucleotide sequence of the domestic dog (*Canis familiaris*) mitochondrial genome. Mol. Biol. Evol..

[42] Song J.J., Wang W.Z., Otecko N.O., Peng M.S., Zhang Y.P. (2016). Reconciling the conflicts between mitochondrial DNA haplogroup trees of *Canis lupus*. Forensic Sci. Int. Genet..

[43] Peng M.S., Fan L., Shi N.N., Ning T., Yao Y.G., Murphy R.W., Zhang Y.P. (2015). DomeTree: A canonical toolkit for mitochondrial DNA analyses in domesticated animals. Mol. Ecol. Resour..

[44] Malmstrom H., Stora J., Dalen L., Holmlund G., Gotherstrom A. (2005). Extensive human DNA contamination in extracts from ancient dog bones and teeth. Mol. Biol. Evol..

[45] Tsuda K., Kikkaw Y., Yonekawa H., Tanabe Y. (1997). Extensive interbreeding occurred among multiple matriarchal ancestors during the domestication of dogs: Evidence from inter-and intraspecies polymorphisms in the D-loop region of mitochondrial DNA between dogs and wolves. Genes Genet. Syst..

[46] Marinov M., Teofanova D., Gadjev D., Radoslavov G., Hristov P. (2018). Mitochondrial diversity of Bulgarian native dogs suggests dual phylogenetic origin. PeerJ.

[47] Achilli A., Olivieri A., Pala M., Metspalu E., Fornarino S., Battaglia V., Accetturo M., Kutuev I., Khusnutdinova E., Pennarun E. (2007). Mitochondrial DNA variation of modern Tuscans supplementary orts the near eastern origin of Etruscans. Am. J. Hum. Genet..

[48] Fregel R., Suárez N.M., Betancor E., González A.M., Cabrera V.M., Pestano J. (2015). Mitochondrial DNA haplogroup phylogeny of the dog: Proposal for a cladistic nomenclature. Mitochondrion.

[49] Losey R.J., Garvie-Lok S., Leonard J.A., Katzenberg M.A., Germonpré M., Nomokonova T., Savel’ev N.A. (2013). Burying dogs in ancient Cis-Baikal, Siberia: Temporal trends and relationships with human diet and subsistence practices. PLoS ONE.

[50] Bartholdy B.P., Murchie T.J., Hacking K., Verwoerd C. (2017). Dog days on the Plains: A preliminary aDNA analysis of canid bones from southern Alberta and Saskatchewan. Can. J. Archaeol..

