# Supplementary material for: Evidence for Early European Neolithic Dog Dispersal: New Data on Southeastern European Subfossil Dogs from the Prehistoric and Antiquity Ages
Source: Genes (Basel). 2019 Sep 26;10(10):757. doi: 10.3390/genes10100757 (PMC6826387; doi:10.3390/genes10100757)
Supplement: Supplementary file 1 [file genes-10-00757-s001.zip › Supplementary Figure S1 Sample locations.docx]

**Supplementary Figure S1**. Map showing sample locations. Mitochondrial DNA haplogroup distributions in subfossil dogs from Bulgaria are presented.


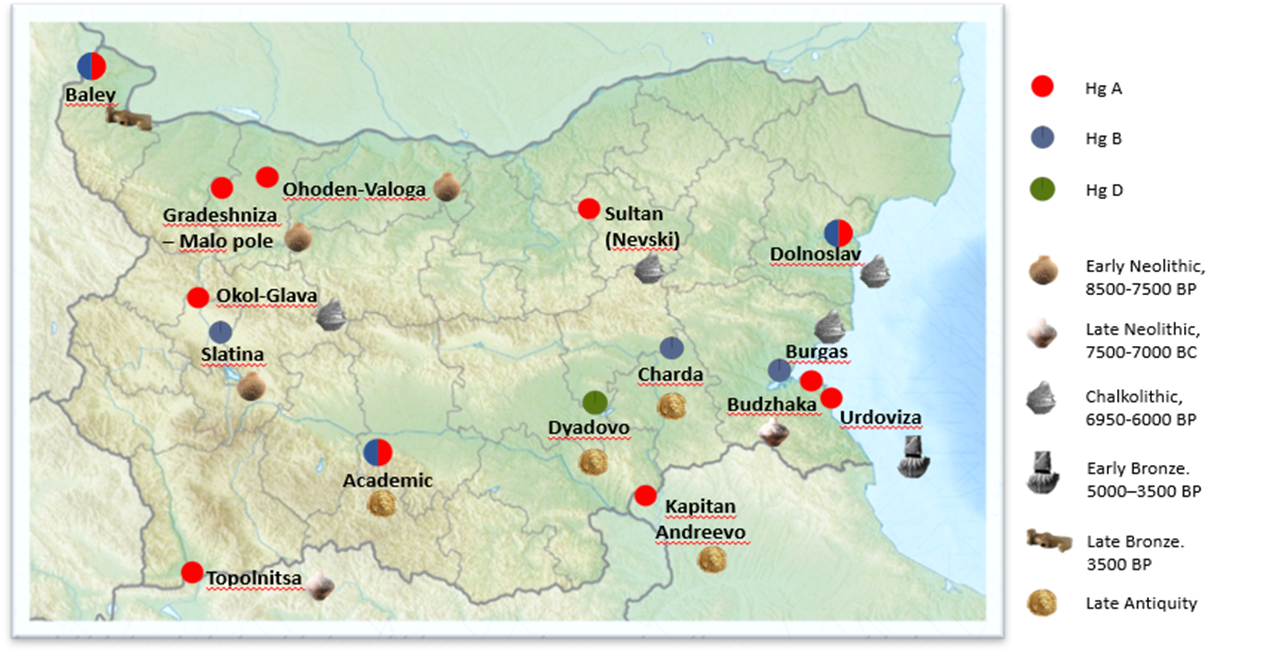


**Table S2.** Primers used for amplification of HVR1 from the D-loop region of mtDNA. The position of the primers is based to the reference sequence NC_002008 (Kim et al., 1998).

| **Region** | **Primer**  **sense/antisense** | **Sequence (primer length)**  **5'-3'** | **Reference** | **Product size, bp** |
| --- | --- | --- | --- | --- |
| I.^a^ | S_A 15341 | ttaccttggtcttgtaaacc(20) | ^1^ | 388 |
|  | 15728AS | gggacattacgagcaagggttgatgg (26) | own design |  |
| I.^a^ | 15341-s | ttaccttggtcttgtaaacc(20) | own design | 363 |
|  | HV1B2-R 15703 | gtttcttgtttctcgaggcatggtgat (27) | ^2^ |  |
| I.^a,b^ | 15433S | gagattcttcttaaactattccctg (25) | own design | 296 |
|  | 15728AS | gggacattacgagcaagggttgatgg (26) | own design |  |
| I.^c^ | HV1B2-F 15527 | ccctcccctatgtacgtcg (19) | ^2^ | 177 |
|  | HV1B2-R 15703 | gtttcttgtttctcgaggcatggtgat (27) | ^2^ |  |
| I.^b,c^ | 15433S | gagattcttcttaaactattccctg (25) | own design | 270 |
|  | HV1B2-R 15703 | gtttcttgtttctcgaggcatggtgat (27) | ^2^ |  |
| II.^a,b^ | 15651S | cgaatgcatatcacttagtcc (21) | own design | 245 |
|  | HV1C2-R 15895 | gtttcttttatgtgtgatcatgggctga (28) | ^2^ |  |
| II.^c^ | HV1C2-F 15680 | cttaatcaccatgcctcgaga (21) | ^2^ | 125 |
|  | AS_A 15804 | ctgaagtaagaaccagatgcc (21) | ^1^ |  |
| III.^a,b^ | S_B 15746 | catactaacgtgggggttac (20) | ^1^ | 362 |
|  | AS_B 16107 | ccattgactgaatagcacctt (21) | ^1^ |  |
| III.^c^ | 15781S | cctggcatctggttcttacttc (22) | own design | 310 |
|  | 16090AS | ccttgattttatgcgtgagttg (22) | own design |  |

**Abbreviations**: ^a^ negative blank control; ^b^ first nested PCR amplification and negative blank control; ^c^ second nested PCR amplification and sequencing primer.

1. Brown, S. K. Darwent, C. M. & Sacks, B. N. Ancient DNA evidence for genetic continuity in arctic dogs. *Journal of Archaeological Science* 40, 1279-1288 https://doi.org/10.1016/j.jas.2012.09.010 (2013).
2. van Asch, B. *et al.* Forensic analysis of dog (*Canis lupus familiaris*) mitochondrial DNA sequences: an inter-laboratory study of the GEP-ISFG working group. *Forensic Science International: Genetics* 4, 49-54 https://doi.org/10.1016/j.fsigen.2009.04.008 (2009).
