# Supplementary material for: Evidence for Early European Neolithic Dog Dispersal: New Data on Southeastern European Subfossil Dogs from the Prehistoric and Antiquity Ages
Source: Genes (Basel). 2019 Sep 26;10(10):757. doi: 10.3390/genes10100757 (PMC6826387; doi:10.3390/genes10100757)
Supplement: Supplementary file 1 [file genes-10-00757-s001.zip › Supplementary Table S1 Archaeological samples .docx]

**Supplementary Table S1**. Archaeological samples studied, with associated codes, skeletal element used, and additional information supplied. Site details and dates are given above each set of specimens. Archaeological dates are based on the context of, and evidence from, each excavation. Phenotype versus mitotype and maximum mitochondrial region sequence lengths are included.

| **Cultural Phase /SE European cultures** | **Settlement** | **Archaeological code** | **Sequence_ID** | **Bone Element** | **Max sequence length (bp)** | **Haplogroup** | **Species** | **Site references** |  |
| --- | --- | --- | --- | --- | --- | --- | --- | --- | --- |
| **Early Neolithic**  **Karanovo I-II, 8500-7500 BP** | | | | | | | | | |
| Karanovo II  Vinca- Gradeshniza | Slatina, Sofia district | 11/S792 | Slat-11 | mandibula | **564** | **B** | C. f. palustris-intermedius (vlasac)?? |  |  |
|  |  | 12/S793 | Slat-12 | mandibula | n/a | n/a | *C. f. palustris-intermedius (vlasac)??* |  |  |
|  | Ohoden–Valoga  (8500-8000 BP) | 18 | Ohod-18 | humerus | n/a | n/a | *C. familiaris* |  |  |
|  |  | 19 | Ohod-19 | humerus | **564** | **A** | *C. familiaris* |  |  |
|  | Gradeshniza – Malo pole | 13/S815 | MPGra-13 | mandibula | **564** | **A** | *C. f. palustris-intermedius (vlasac)??* | 1  2 |  |
| **Late Neolithic**  **Karanovo II-III, 7500-7000 BP** | | | | | | | | | |
| Topolnica-Akropotamos | [Topolnica,](https://issuu.com/ivanvajsov/docs/promachon-topolnica__offprint_)  Promachon  (7200-6600 BP) | 1 | ToPro-1 | mandibula | **696** | **A** | *C. f. palustris* | 3 |  |
|  |  | 6/S980 | ToPro-6 | mandibula | n/a | n/a | *C. f. palustris* | 3 |  |
|  |  | 7/S977 | ToPro-7(I) | mandibula | **177** | **A** | *C. f. palustris* | 3 |  |
| Gumelnitsa-  Kodjadermen- | Budzhaka-Sozopol (7200-7000 BP) | 4 | Budz-4(II) | mandibula | **216** | **A** | *C. f. palustris* | 4,10,12 |  |
|  |  | 5 | Budz-5 | dens | n/a | n/a | *C. f. palustris* | 4,10,12 |  |
| **Early Chalcolithic**  **Karanovo III-IV, 6950-6500 BP** | | | | | | | | | |
|  | Okol‐Glava, Gniljane village, Sofia district | 10/S794 | OkGl-10 | mandibula | **528** | **A** | *C. f. palustris-intermedius (vlasac)??* |  |  |
|  | Sultan (Nevski) Popovo | 9/S828 | SulPo-9 | mandibula | **351** | **A** | *C. f. palustris-intermedius* | 5,11 |  |
| Gumelnitsa-Kodjadermen-Karanovo | Settlement mound Burgas |  | Burg-22(I,III) | dens | **496** | **B** |  | 4,10,12 |  |
| **Late Chalcolithic**  **Karanovo VI, 6500-6000 BP** | | | | | | | | | |
| Hamangia | Dolnoslav, Varna | 2/S694 | Doln-2(I,III) | mandibula | **489** | **A** | *C. f. palustris* | 6 |  |
|  |  | 3/S695 | Doln-3 (III) | mandibula | **411** | n/a | *C. f. palustris-* | 6 |  |
|  |  | 8/S712 | Doln-8 | mandibula | **564** | **B** | *C. f. palustris-intermedius* | 6 |  |
| **Early Bronze**  **6000-5000 BP** | | | | | | | | | |
| Thrace | Urdoviza (Kiten) | 14/U6 | Urdo-14 | mandibula | **564** | **A** | *C. f. palustris-intermedius* | 7,12 |  |
| **Late Bronze**  **6000-5000 BP** | | | | | | | | | |
| Thrace | Baley |  | Bal-16 | mandibula | **564** | **A** | *C. f. intermedius* | 8 |  |
|  |  |  | Bal-17 | mandibula | **310** | **B** | *C. f. intermedius* | 8 |  |
| **Late antiquity** | | | | | | | | | |
| Thrace-Roman Empire | Kapitan Andreevo |  | KpAn-15 | mandibula | **564** | **A** | *C. f. intermedius* |  |  |
| Thrace-Roman Empire | Dyadovo village - Nova Zagora |  | DyaNZ-23 | dens | **564** | **D** | *C. familiaris* | 9 |  |
| Thrace-Roman Empire | Charda village, Yambol district |  | Char-24 | vertebra | **549** | **B** | *C. f. palustris-intermedius (vlasac)??* |  |  |
| Thrace-Roman Empire | Trakia motorway, Yambol district |  | TakY-25 | dens | 502 | **A** | *C. f. palustris-intermedius (vlasac)??* |  |  |
| Thrace-Roman Empire | Academic, Plovdiv | 20/S989 | Acad-20 | mandibula | 411 | **A** | *C. familiaris* |  |  |
|  |  | 20/S990 | Acad-21 | mandibula | 411 | **B** | *C. familiaris* |  |  |

**References**

1. Nikolov, B. Gradeshnitsa. Nauka i Izkustvo Publ. House Sofia. (1974)
2. Todorova, H. & Vajsov, I. The Neolithic epoch in Bulgaria. Nauka i Izkustvo Publ. House. Sofia, р. 287. (1993).
3. Bojadziev, J. Absolute chronology of the Neolithic and Eneolithic cultures in the valley of Struma.The Struma/Strymon river valley in prehistory. In the Steps of James Harvey Gaul **2**, 309-316. (2007).
4. Nikolov, V. A reinterpretation of Neolithic complexes with dug-out features: pit sanctuaries. Studia praehistorica, **14**, 91-119. (2011).
5. Klimscha, F. Flint axes, ground stone axes and “battle axes” of the Copper Age in the Eastern Balkans. DAVIS, V.; EDMONDS, M. Stone axe studies III. Oxford: Oxbow, 361-382. (2011).
6. Raduncheva A. The temple centre near Dolnoslav village, Plovdiv distr. And some aspects of the rituals at the end of the Chalcolithic Epoch. New Bulgarian University & Archaeological Inst. and museum at the Bulg. Acad. Sciences. Annuary of the Departmernt of Archaeology. Sofia, II-III, pp. 168-181. (1996).
7. Spassov, N. Hristova, L. & Iliev, N. The domesticated horses from the submerged prehistoric village of Urdoviza (Kiten) on the Bulgarian Black Sea coast–among the oldest known. Historia naturalis bulgarica 25, 11-14. (2018).
8. Yotzova A., Alexandrov S., Hristova T. & Ivanov G. (2012) The Late Bronze Age Settlement Baley (North-West Bulgaria). Stratigraphy and Features. Archaeology. 53, 7-26
9. Nikolov, V., Bacvarov, K., Popov, H., & Forschungen, I. 2011. Archaeological evidence for paleoclimate change on the southern Bulgarian Black Sea coast from the sixth to third millennium BC. Interdisziplinäre Forschungen zum Kulturerbe auf der Balkanhalbinsel, 131.
10. Bojadžiev J. (2002) Die Absolute Chronologie der neo-und aneolitishen Graberfelder von Durankulak. In: (ed. By H. Todorova). Durankulak. Band II, Teil1. Die Prahistorischen Graberfelder. Deutshes archaologisches Institut in Berlin. Sofia, pp. 67-69.
11. Ivanov S. (1959) The food with animal origin of the inhabitants of the Southern Gate of Preslav. Archaeological Institute and Museum, 22, p. 208 p. Note: The material is from the unpubl. Coll. of S. Ivanov (1957-1959, labeled IX-X century).
12. Leshtakov P. & Klasnakov M. (2010) Rescue Archaeological Excavations of the Praehistoric Site in the region of Acladi Cheiri near Chernomorets Village. Archeologicheski otkritia I razkopki prez 2009 (Archaeological discoveris and excavations in 2009). Natl. Archaeological Inst. And Museum,Bulg. Acad Sci Sofia, 2009, pp 58-61.
